# *Salmonella* Vitkin Outbreak Associated with Bearded Dragons, Canada and United States, 2020–2022

**DOI:** 10.3201/eid3002.230963

**Published:** 2024-02

**Authors:** Katherine Paphitis, Caroline A. Habrun, G. Sean Stapleton, Alexandra Reid, Christina Lee, Anna Majury, Allana Murphy, Heather McClinchey, Antoine Corbeil, Ashley Kearney, Katharine Benedict, Beth Tolar, Russell O. Forrest

**Affiliations:** Public Health Ontario, Toronto, Ontario, Canada (K. Paphitis, C. Lee, A. Majury, A. Murphy, A. Corbeil);; Centers for Disease Control and Prevention, Atlanta, Georgia, USA (C.A. Habrun, G.S. Stapleton, K. Benedict, B. Tolar);; Ontario Ministry of Agriculture, Food, and Rural Affairs, Guelph, Ontario, Canada (A. Reid);; Ontario Ministry of Health, Toronto (H. McClinchey);; Public Health Agency of Canada, Winnipeg, Manitoba, Canada (A. Kearney);; Public Health Agency of Canada, Guelph (R.O. Forrest)

**Keywords:** Salmonella enterica serovar Vitkin, bacteria, infections, outbreak, bearded dragons, reptiles, whole-genome sequencing, serovar, zoonoses, Canada, United States

## Abstract

We identified 2 cases of *Salmonella* *enterica* serovar Vitkin infection linked by whole-genome sequencing in infants in Ontario, Canada, during 2022. Both households of the infants reported having bearded dragons as pets. The outbreak strain was also isolated from an environmental sample collected from a patient’s bearded dragon enclosure. Twelve cases were detected in the United States, and onset dates occurred during March 2021–September 2022 (isolates related to isolates from Canada within 0–9 allele differences by core-genome multilocus sequence typing). Most US patients (66.7%) were <1 year of age, and most (72.7%) had reported bearded dragon exposure. Hospitalization was reported for 5 (38.5%) of 13 patients. Traceback of bearded dragons identified at least 1 potential common supplier in Southeast Asia. Sharing rare serovar information and whole-genome sequencing data between Canada and the United States can assist in timely identification of outbreaks, including those that might not be detected through routine surveillance.

Nontyphoidal *Salmonella* are consistently among the most commonly reported bacterial pathogens linked to enteric illness in Canada and the United States (*1**,**2*). In most cases, human infection is ultimately attributed to consumption of specific foods or to contact with animals (*1**,**3**,**4*). Persons usually show development of illness within 12–36 hours after exposure (range 6 hours–16 days), and most recover without treatment (*5**,**6*). However, children <5 years of age, adults >65 years of age, and persons with weakened immune systems are more likely to show development of invasive infections (*5**–**7*).

There are >2,600 serovars of *Salmonella*, more than half of which belong to subspecies I (*S*. *enterica* subsp. *enterica*) and can cause human and animal illness (*8**–**10*). Although many *Salmonella* serovars are predominantly associated with reptiles and amphibians, most of these have low-to-moderate zoonotic potential and are believed to account for <1% of human salmonellosis, mainly affecting persons with weakened immune systems and small children (*11*). The burden of *Salmonella* caused by specific serovars varies by country and region, partially because of the distribution of reservoir animal species and because of local food preparation and dietary preferences. Although some serovars, such as Typhimurium and Enteritidis, are globally distributed, others, such as Heidelberg and Newport, are more commonly found in North America (*10**,**12*).

In June 2022, Public Health Ontario (PHO) noted 2 cases of a rare *Salmonella* serovar (*Salmonella enterica* serovar Vitkin [*Salmonella* Vitkin]) reported by a local public health unit. The cases were closely related (within 2 alleles based on core-genome multilocus sequence typing [cgMLST]), and onset dates were 4 weeks apart, suggestive of a common exposure.

No cases of *Salmonella* Vitkin were reported in Ontario or elsewhere in Canada for at least 14 years before these 2 cases (the extent of the data available for review), and the Vitkin serovar had only been seen 23 times in PulseNet USA (https://www.cdc.gov/pulsenet/index.html) before 2021 (*13*). Similarly, no outbreaks associated with *Salmonella* Vitkin were documented within the US Centers for Disease Control and Prevention (CDC) National Outbreak Reporting System during 1971–2020 (*14*).

Given the rarity of this serovar in North America, the Public Health Agency of Canada (PHAC) coordinated communication between PHO and CDC to explore whether CDC had recently identified any cases of this rare serovar in the United States. CDC confirmed that several US isolates of *Salmonella* Vitkin had been reported to PulseNet USA by multiple US states over the previous year. Analysis of whole-genome sequencing data showed that isolates from the United States and Canada were related within 0–9 cgMLST allele differences. We report the multinational outbreak investigation performed by PHO, PHAC, CDC, and partner agencies to identify the source of this rare outbreak.

## Methods

### Epidemiology

In Ontario, public health units use standardized questionnaires for case interviewing and data collection (*15*). Using the questionnaire for *Salmonella* spp., local investigators interviewed a proxy respondent (parent or guardian) for each case and collected data on symptoms and relevant exposures during the 7 days before symptom onset. PulseNet Canada cluster codes for *Salmonella* spp. are assigned when >2 isolates occur within a 60-day period and are related within 0–10 alleles by whole-genome multilocus sequence typing (wgMLST) (*16*).

The range of 0–10 wgMLST allele differences might expand or narrow during an investigation on the basis of available laboratory, epidemiologic, and traceback evidence. Wider allele ranges have been previously observed in *Salmonella* outbreaks linked to zoonotic sources (*17**,**18*). Ontario case-patients were defined as patients infected with laboratory-confirmed *Salmonella* Vitkin who had an isolate matching the PulseNet Canada cluster code (2206VitkinWGS-1ON) by whole-genome sequencing (WGS) and had illness onset during April–September 2022. Data were shared with PHO through the integrated Public Health Information System reporting platform.

In July 2022, PHAC notified CDC of the Ontario case cluster and provided investigators with relevant case demographics, reptile exposure, and laboratory information. CDC queried PulseNet USA, the national molecular subtyping network for foodborne disease surveillance in the United States to confirm relatedness and identify genetically related cases. PulseNet USA cluster codes for *Salmonella* spp. are typically assigned when >7 clinical isolates occur within a 60-day period and are related within 10 alleles by cgMLST.

US case-patients were defined as patients infected with laboratory-confirmed *Salmonella* Vitkin that was genetically related within 0–9 allele differences based on cgMLST who had an illness onset of March 2021–September 2022. State and local public health investigators interviewed patients (or their proxies) about possible food or animal exposures before illness onset; those data were shared with CDC through the System for Enteric Disease Response, Investigation, and Coordination platform (*19*). In September 2022, CDC requested that state health departments collect additional information on bearded dragon husbandry behaviors, purchase locations, and feed.

### Laboratory Investigation

**Figure 1:**
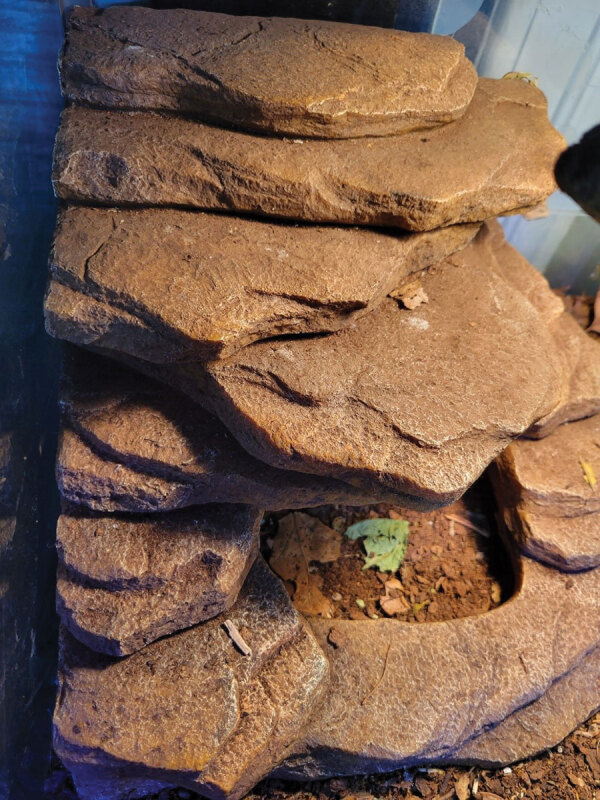
Bearded dragon rock cave, source of the environmental isolates that were closely related to the outbreak strain of *Salmonella* *enterica* serovar Vitkin found in patients in Ontario, Canada, 2022. The environmental isolates were within 1 and 2 alleles by core-genome multilocus sequence typing to those from the patients.

**Figure 2:**
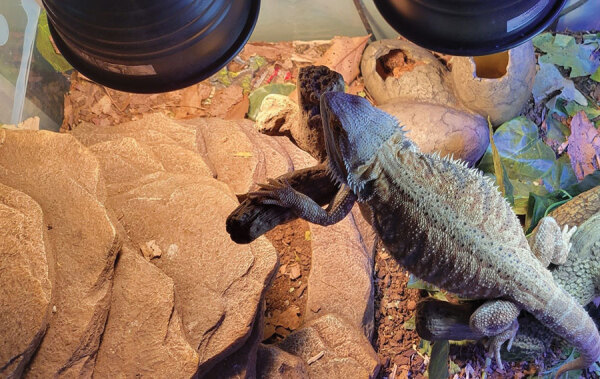
Female bearded dragon belonging to household of 1 of 2 case-patients infected with *Salmonella* *enterica* serovar Vitkin, Ontario, Canada, 2022.

We analyzed 4 environmental swab specimens collected from the bearded dragon enclosure in 1 Ontario case household (Figure 1) and a fresh fecal specimen (collected in July 2022) from the bearded dragon (Figure 2) by using routine aerobic culture at the PHO laboratory (*20*). We also analyzed open samples of dried, pelleted reptile feed and a reptile calcium supplement by routine culture at the PHO laboratory (*20*). PHO runs WGS for all *S. enterica* by using the standardized PulseNet Canada protocol (*21*). WGS data are analyzed locally and then uploaded to a centralized BioNumerics version 7.6 (bioMérieux, https://www.biomerieux.com) database, where it is compared with data from other jurisdictions in Canada to identify multijurisdictional clusters by using wgMLST. PulseNet Canada uses wgMLST as a primary method for identifying genetically related isolates but also has the ability to use cgMLST for comparison with other jurisdictions that use this method. Canada and the United States routinely share genomic data under a bilateral information sharing agreement for comparison between the 2 countries because Canada does not yet upload WGS data to a public repository, such as the National Center for Biotechnology Information (NCBI; https://www.ncbi.nlm.nih.gov), in real time.

In this investigation, sequence data from a representative *Salmonella* Vitkin isolate was requested from PulseNet USA and added to the Canada database, where it was determined to be related to the Ontario cases within 1–3 cgMLST allele differences. A representative sequence from Canada was shared with PulseNet USA for comparison by cgMLST to identify related isolates in the United States. For Canada isolates, WGS data were deposited retrospectively in NCBI in the PulseNet Canada Salmonella BioProject (PRJNA543337). US sequences were uploaded to NCBI under the PulseNet *Salmonella* BioProject (PRJNA230403).

We reviewed veterinary isolate data, representing specimens collected from sick or deceased reptiles and tested by the Animal Health Laboratory (AHL) in Ontario during 2013–2022 to determine whether *Salmonella* Vitkin had been detected in reptiles in Ontario by this surveillance program. Reptile species tested at the AHL reflect common pet species as well as samples from private zoologic institutions. No US patients permitted investigators to screen their bearded dragons for *Salmonella* spp.

### Traceback of Reptiles

We conducted a traceback investigation for each of the patient’s bearded dragons to identify a potential common supplier or breeder. Where applicable, local pet stores, and intermediary suppliers were interviewed to obtain information on the source(s) of their reptiles and whether they were acquired by local breeders or imported.

### Ethics

This study did not require research ethics committee approval because activities described herein were conducted in fulfillment of the legislated mandate of PHO “to provide scientific and technical advice and support to the health care system and the Government of Ontario in order to protect and promote the health of Ontarians” and are therefore considered public health practice, not research (*22*). Similarly, this activity was conducted consistent with applicable federal law and CDC policy (see e.g., 45 C.F.R. part 46, 21 C.F.R. part 56; 42 U.S.C. §241(d); 5 U.S.C. §552a; 44 U.S.C. §3501 et seq.).

## Results

### Epidemiology

Both Ontario case-patients were <1 year of age and had isolates related within 3 alleles. Onset dates were reported to be ≈4 weeks apart, in April and May 2022. No hospitalization or death was reported for either of the Ontario patients.

We did not identify any common food exposures (including infant formula) among the Ontario patients. However, the proxy respondent for each patient reported >1 bearded dragons in the household (Table). We noted no commonalities between households with respect to bearded dragon diet. One household reported feeding their reptile(s) feeder mice.

**Table:** Patient demographics for 14 *Salmonella enterica* serovar Vitkin cases in Canada and the United States, March 2021–October 2022*

Characteristic	Value
Median age, y	<1 (range <1–28)
Female sex	8 (57.1)
Contact with bearded dragons†	10 (76.9)
Hospitalization	5 (38.5)

*Values are no. (%) except as indicated. Of the 14 cases, 2 were reported in Canada and 12 in the United States. †Of 13 cases with exposure information available.

**Figure 3:**
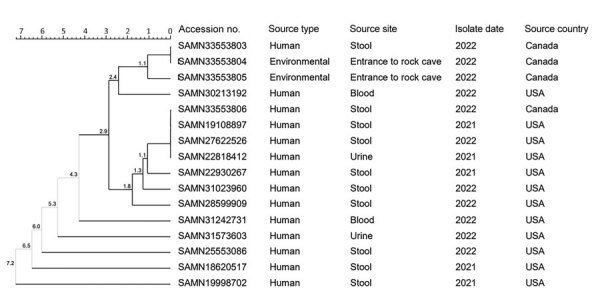
Unweighted pair group method with arithmetic mean dendrogram of core-genome multilocus sequence typing results for human and environmental isolates included in the Canada and the United States *Salmonella* *enterica* serovar Vitkin outbreak investigation, 2020–2022. Tree was generated by using BioNumerics version 7.6 (bioMérieux, https://www.biomerieux.com). Numbers along branches are median allele differences. Shown are GenBank accession numbers.

**Figure 4:**
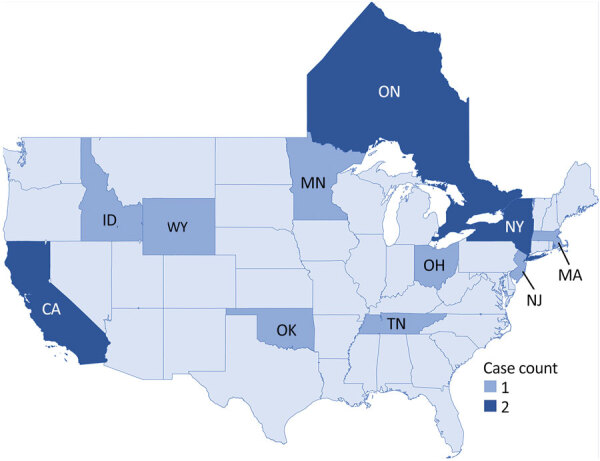
*Salmonella* *enterica* serovar Vitkin infection case counts, by state (United States) and province (Canada), 2021–2022.

Twelve cases of *Salmonella* Vitkin were identified in the United States, were considered genetically related to the outbreak strain, and had illness onset dates ranging from March 2021 to September 2022 (Figure 3). The US cases were identified across 10 states (Figure 4). Of 11 patients with additional information available, 5 (45%) were hospitalized and no deaths were reported. The median age of US case-patients was the same as that of the Ontario case-patients, including 8 (67%) children <1 year of age (Table).

Among the 11 US patients who had available exposure information, 8 (73%) reported either having direct contact with bearded dragons or having one in their household (Table). No common food exposures were identified among patients.

### Laboratory Investigation

One environmental swab specimen, collected from the entrance to the bearded dragon’s rock cave (Figure 2), was positive for *Salmonella* Vitkin. Two isolates from this swab specimen were related to the outbreak strain by WGS (Figure 3). *Salmonella* spp. were not detected from the other 3 environmental swab specimens, the fresh fecal specimen collected from the same reptile enclosure, the open sample of reptile feed, or the reptile calcium supplement. Canada human isolates and 2 environmental isolates were genetically related within 0–3 cgMLST allele differences; they also were genetically related within 0–9 cgMLST allele differences to 12 US clinical cases. The 4 Canada *Salmonella* Vitkin isolates linked to this investigation were the only Vitkin isolates in the Canada database. Therefore, no additional strains were analyzed outside the US isolates.

### Traceback of Reptiles

Ontario patients reported purchasing bearded dragons from 2 different pet store locations in Ontario, and US patients reported purchases from 4 different US pet store locations and an online source. Both Ontario pet stores were supplied by a single common intermediary supplier, which imported bearded dragons from various suppliers, including an international supplier located in Southeast Asia. This intermediary supplier reported that they did not ship bearded dragons from or to the United States and that they had ceased importing reptiles from the international supplier in late 2021. The US pet stores were supplied by 1 of 3 common intermediary suppliers, 1 of which purchased bearded dragons from the same international supplier. No single breeder was identified as the source for all bearded dragons implicated in this investigation.

### Reptile Isolate Data

A review of reptile veterinary isolate data compiled by the AHL found that, although most of the specimens tested from 2013–2022 were from bearded dragons (by reptile species, 21.3%, n = 13), *Salmonella* Vitkin was not isolated from any of these, or from any other reptile species. The serovars most commonly isolated from bearded dragons were *Salmonella* Amsterdam (23.1%, n = 3) and *Salmonella* Kisarawe (23.1%, n = 3).

### Public Health Response

Local public health investigators visited each pet store identified by the Ontario case-patients as the location of bearded dragon purchase and provided pet store operators with fact sheets summarizing information on *Salmonella* infection and prevention associated with reptiles for distribution to future customers. Information provided by fact sheets included recommendations for pet owners to wash their hands after handling reptiles, to clean and disinfect any surfaces that come into contact with their reptile, and to supervise children during interactions with reptiles, while noting that these animals are not recommended as pets for households with >1 persons at increased risk for severe illness (*23*). Canada and US public health partners shared their outbreak findings with public health officials located in the country of the common Southeast Asia bearded dragon supplier, as well as educational resources on preventing *Salmonella* transmission from bearded dragons.

CDC first communicated to the public regarding this outbreak on October 18, 2022. The website post highlighted investigation details and information for actions to take to minimize the risk for reptile-associated *Salmonella* infections (*24*). Similar general preventative information in the form of a public-facing factsheet is available on the PHAC webpage (*23*). The investigations in Canada and the United States were closed in December 2022.

## Discussion

*Salmonella* Vitkin infections linked to this outbreak disproportionately affected infants and often resulted in severe illness, as shown by 78% of reported case-patients being <1 year of age and almost 40% of patients reporting hospitalization because of illness. Contact with bearded dragons was the source of this outbreak. Reporting of indirect or direct contact with bearded dragons by most case-patients and isolation of the same serovar from a reptile enclosure supported bearded dragons as the most likely source of exposure. Many infants likely were indirectly exposed to infection by the contaminated clothing or hands of caregivers or by contact with contaminated environmental surfaces within the home.

Reptiles, including bearded dragons, lizards and snakes, are among several animal species that are becoming more common in Canada and elsewhere as household pets (*25**–**27*). Reptiles can carry *Salmonella* spp. in their gastrointestinal tract without displaying signs of illness and can intermittently shed the bacteria in their feces (*28*). Fecal shedding can be prompted or exacerbated by stressors, such as handling, transportation, or illness (*28*). Persons might become ill with *Salmonella* infections if they have direct or indirect contact with reptiles or their environment, particularly if they do not wash their hands after handling or caring for their reptiles or if they do not clean and disinfect contaminated surfaces (*26**,**29*).

A study found that, although most cases of human salmonellosis in Ontario during 2010–2012 were attributed to foodborne transmission, 35.5% (n = 107) of reported cases were attributed to contact with reptiles or amphibians (*3*). This finding is notable because, according to the most recent Canadian Foodbook Report, produced by the Public Health Agency of Canada in 2015 after a population-based telephone survey of the Canadian population, only 2.1% of Ontario respondents reported having any contact with reptiles in the previous 7 days, indicating the relatively small proportion of the population who might keep these reptiles as pets (*30*). In comparison, 31.0% of Ontario respondents reported having contact with a cat and 42.2% reported having contact with a dog during the same time period (*30*). Similarly, a recent national pet owner survey found that ≈90.5 million US homes reported owning >1 pet. Among households that owned a pet, ≈6% (5.7 million) owned a reptile, compared with 76% (69.0 million) that owned a dog and 50% (45.3 million) that owned a cat (*31*).

Captive bearded dragons have higher rates of *Salmonella* carriage compared with those in the wild, and stress can increase the frequency of *Salmonella* shedding (*32*). Awareness and consideration of steps that can be taken by owners and breeders of bearded dragons to reduce stressors (such as avoiding overcrowding during transport, and provision of adequate space and enrichment items within enclosures) could theoretically reduce *Salmonella* shedding and subsequent human exposure (*33*). Bearded dragon owners should be encouraged to restrict roaming of their bearded dragons outside of their enclosure to surfaces and items that are able to be cleaned and disinfected afterwards.

If reptile owners are not aware that their reptiles can carry and shed *Salmonella* spp., they might fail to take appropriate preventive measures, increasing the risk for illness among household members, including those who do not have direct contact with reptiles. Because some *Salmonella* serovars can survive on surfaces for several days to months, surfaces might serve as a source of indirect exposure if they are not cleaned and disinfected after coming into contact with bearded dragons or other reptiles (*34**,**35*). Previous studies have noted that *Salmonella* carriage is common among captive reptiles and that a high-density population of animals (e.g., in breeding facilities, during transport or at point of sale) can promote the transfer of *Salmonella* spp. between reptiles, particularly if the reptiles are fed with rodents (*26**,**36*).

There have been several reported outbreaks of *Salmonella* infections linked to contact with reptiles in Canada and the United States, including outbreaks specifically linked to bearded dragons. Those outbreaks have involved several different serovars, including Uganda (2022), Muenster (2020), Apapa (2018), and Cotham (2014) (*27*,*37**–**40*). Although those outbreaks affected persons of all age groups, children were overrepresented.

To date, there has been minimal published literature on *Salmonella* Vitkin infections in humans; we identified only 1 article summarizing a case of meningitis caused by *Salmonella* Vitkin infection in an infant (1 month of age) after exposure to a pet turtle (*29*). Children, particularly those <5 years of age, might be at increased risk for exposure to *Salmonella* infections and other enteric infections because of poor hand hygiene, developing immune systems, and a tendency to mouth objects (*41*). Children might also be more susceptible to less virulent strains of the bacteria, which might explain the relatively young median age of those involved in reported outbreaks involving reptiles (*25*). Because transmission can occur by direct and indirect contact with reptiles, parents might not recognize indirect reptile contact as a risk. As such, they might fail to take appropriate preventive measures, including keeping children away from the reptiles and any potentially contaminated surfaces. Furthermore, parents and caregivers might not think to change potentially contaminated clothing or wash their hands between handling reptiles and interacting with children (*11*). Infants could be at increased risk for indirect exposure by the clothing of adult household members because they are more likely to be held or carried than independently mobile toddlers and older children.

Although a fresh fecal specimen from 1 bearded dragon was collected during the *Salmonella* Vitkin investigation and found to be negative for *Salmonella* spp., this specimen was collected 3 months after the onset of illness in the child within the same household, and the bearded dragon might have no longer (or only intermittently) been shedding *Salmonella* spp. in its stool at the time. A previous study of household *Salmonella* transmission associated with pet reptiles in Germany found that in 15 (78.9%) of 19 households with a child with confirmed *Salmonella* infection, an identical serovar was confirmed in both the infected case and >1 reptile in the household (*42*). The authors further noted that, although reptiles might be simultaneously colonized with multiple *Salmonella* serovars, shedding may be intermittent, and a negative cloacal or fecal specimen does not mean that the reptile is free from *Salmonella* spp. (*42*). Instead, persons should assume that all reptiles could be carrying *Salmonella* spp. (*42*).

Although the intermediary reptile supplier in Canada in this investigation did not report US importation or exportation of reptiles, it is unknown whether cross-border importation or export of reptiles is common practice or the extent to which importation of reptiles to North America might occur. Ontario has no record-keeping requirements for persons who breed, import, export or sell reptiles. Bearded dragons within the pet trade are entirely maintained by captive breeding operations that might operate at a small scale (i.e., a person with 2 bearded dragons) or at a commercial scale (*33*). The stress and close confinement during transport is associated with an increased risk for *Salmonella* shedding and transmission (*11*). Although cases of *Salmonella* Vitkin infection linked to this outbreak were reported in the United States until November 2022, no additional cases were reported in Ontario (or elsewhere in Canada) after the intermediary supplier in Canada ceased importing bearded dragons from the common international breeder that was identified in this investigation.

Because reptile supply chains in Canada and the United States might be integrated for some species, communication between international public health investigators can assist in identification of multijurisdictional outbreaks associated with reptiles and can help to identify a potentially causative exposure, particularly in situations with rare serovars. Furthermore, environmental sampling can provide microbiological evidence to confirm source identification.

Providing reptile owners with information on the risks of *Salmonella* infection associated with reptiles at the point of purchase/acquisition could support informed decisions about pet choices and necessary precautions. Information could include steps that can be taken to minimize the risk for infection transmission from reptiles to humans, including details on persons who might be at increased risk for illness if exposed and who should ideally avoid reptile contact (*43*). In particular, potential reptile owners who have children <5 years of age should be aware that reptile ownership or contact is discouraged for this age group because, although handwashing and environmental disinfection can reduce the risk for *Salmonella* transmission from reptiles to humans, the increased susceptibility of children to infection and the risk for severe illness if infected make these high-risk pets. Further education for persons and businesses involved in reptile breeding, distribution, and sale could also focus on the need for preventive veterinary care, biosecurity, and environmental cleaning practices (*43*).

Record-keeping requirements for persons involved in the breeding, distribution, and sale of reptiles would assist in traceback investigations and support investigators in identifying the source of infection during outbreak investigations (*43*). Timely collaboration and information sharing can assist in identifying the potential source of a multijurisdictional outbreak, enabling rapid rollout of public health interventions and dissemination of information to the public to prevent illnesses.
